# Effect of Antihypertensive Therapy with Alpha Methyldopa on Levels of Angiogenic Factors in Pregnancies with Hypertensive Disorders

**DOI:** 10.1371/journal.pone.0002766

**Published:** 2008-07-23

**Authors:** Asma Khalil, Shanthi Muttukrishna, Kevin Harrington, Eric Jauniaux

**Affiliations:** 1 The Homerton University Hospital NHS Trust, Queen Mary and Westfield College, University of London, London, United Kingdom; 2 Academic Department of Obstetrics and Gynaecology, UCL Institute for Women's Health, University College London, London, United Kingdom; Khon Kaen University, Thailand

## Abstract

**Background:**

Antihypertensive drugs are believed to lower blood pressure in pre-eclampsia by direct or central vasodilatory mechanisms. However, they could also act by decreasing production of anti-angiogenic proteins involved in the pathophysiology of hypertension and proteinuria in pre-eclampsia (PE). The aim of our study was to evaluate the impact of antihypertensive therapy with alpha methyldopa on maternal circulating levels and placental production of soluble fms-like tyrosine kinase 1 (sFlt-1), soluble endoglin (sEng), vascular endothelial growth factor (VEGF) and placental growth factor (PlGF) in hypertensive disorders of pregnancy.

**Methodology/Principal Findings:**

In a study conducted at University College Hospital and the Homerton University Hospital in London, we recruited 51 women with PE, 29 with gestational hypertension (GH), and 80 matched normotensive controls. Eight (16%) of the women with PE had severe disease. Placental samples were obtained from a further 48 women (14 PE, 10 GH and 24 matched controls). Serum levels of angiogenic factors were measured before and 24–48 hours after commencing antihypertensive therapy with alpha methyldopa for clinical indications. The same parameters were measured in placental extracts. In both PE (*P*<0.0001) and GH (*P*<0.05), serum sFlt-1 was increased and PlGF reduced at all gestations (*P*<0.001) compared to controls. Serum sEng levels were also increased in PE. Placental concentration of sFlt-1 and sEng was significantly higher in women with PE compared to controls and women with GH (*P*<0.0001). The concentration of PlGF was significantly lower in the placental tissue of women with PE compared to GH (*P* = 0.008). Antihypertensive treatment was associated with a significant fall in serum and placental content of sFlt1 and sEng in PE only.

**Conclusions:**

Our data suggest that alpha methyldopa may have a specific effect on placental and/or endothelial cell function in pre-eclampsia patients, altering angiogenic proteins.

## Introduction

Anti-angiogenic and pro-angiogenic factors are known to play an important role in the pathophysiology of pre-eclampsia (PE) [1]–[5]. Studies of maternal serum levels of these factors have shown that soluble endoglin (sEng) and soluble fms-like tyrosine kinase 1 (sFlt-1) are elevated in women presenting with PE whereas vascular endothelial growth factor (VEGF) and placental growth factor (PlGF) are decreased. Some of these changes can be detected several weeks before the appearance of clinical symptoms of PE [6]–[8].

Soluble Flt-1 is a splice variant of VEGF receptor 1 (Flt-1) which is produced by a variety of tissues. Investigation of uterine vein levels of sFlt-1 at cesarean section in pre-eclampsia has suggested a uterine source [9]. The fact that there is a rapid fall in circulating levels of sFlt-1 within 48 hours of delivery is consistent with this concept [4]. Extra-placental sources have also been identified, including endothelial cells, monocytes and peripheral blood mononuclear cells [10]–[12]. Endoglin is a trans-membrane glycoprotein found on cell surfaces highly expressed in endothelial cells and syncytiotrophoblasts [13], [14]. sEng is the soluble form of endoglin found in serum. Its level is increased in the circulation of patients with angiogenic tumours, neovascularisation and myeloid malignancies, and of pregnant women [3]. VEGF and P1GF are vascular endothelial growth factors which are key molecules in angiogenesis and vasculogenesis, in particular during embryogenesis [15]. The main source of VEGF and PlGF during pregnancy is the placental trophoblast. VEGF and PlGF are also expressed in many other tissues, including the villous trophoblast [16]–[25].

The most commonly used drug for the treatment of hypertensive disorders in pregnancy in the UK is alpha methyldopa (αMD). Alpha methyldopa acts on alpha-2 adrenoreceptors and is believed to exert its antihypertensive effect primarily in the central nervous system [26], [27]. Trophoblast cells also possess alpha-2 adrenoreceptors. The activation of these receptors is thought to modulate intracellular messengers such as cyclic AMP (cAMP) [28], [29], so it is possible that αMD also has an effect at this level.

Belgore et al [30] have suggested that, in non-pregnant women with essential hypertension, plasma levels of VEGF and sFlt-1 are elevated compared to normotensive controls, and treatment of hypertension significantly reduces the circulating levels of these molecules. The effect of antihypertensive therapy on trophoblast production and/or release of angiogenic factors and thus on their plasma levels in pregnancy is unknown. The aim of this study was to investigate the effect of antihypertensive therapy with alpha methyldopa on maternal serum concentrations and placental production of sFlt-1, sEng, VEGF and PlGF in pregnant women presenting with hypertensive disorders including pre-eclampsia and gestational hypertension.

## Methods

### Subjects and samples

Serum and placental tissue samples were obtained over an 18 month period from women with singleton pregnancies prospectively recruited in the second and third trimesters of pregnancy at the Homerton University Hospital, London. During this period, approximately 6,000 deliveries took place. Demographic and clinical data including age, body mass index (BMI), parity, blood pressure (BP) and gestational age (GA) were recorded. Gestational age was established on the basis of menstrual date and/or ultrasonographic examination prior to 20 weeks of gestation.

All women were followed up until after delivery, and fetal and maternal outcomes were obtained from the women's medical records and labour ward records. Written consent was obtained from each woman after receiving full written information about the research project. This study was approved by the Camden & Islington Community Local Research Ethics Committee and The University College London Hospitals Committee on the Ethics of Human Research. Exclusion criteria included multiple pregnancy, history of hypertension, diabetes, renal disease or immune disorders or women taking medication which could affect blood pressure.

**Figure 1:**
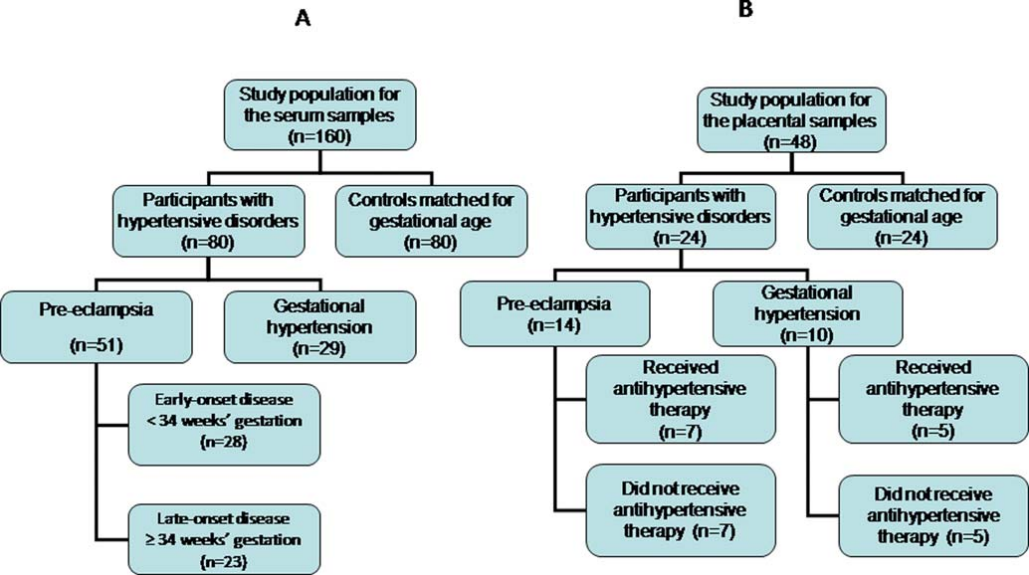
Women recruited to the study. Flow diagrams of women recruited to the serum (a) and placental (b) arms of the study respectively.

The study group in whom serum levels were measured included 51 women presenting with PE, 29 with gestational hypertension and 80 controls (Figure 1a).

Another group of 48 women were recruited for measurement of placental levels: 24 with hypertensive disorders in pregnancy (14 PE, 10 gestational hypertension), and 24 controls matched for maternal age, gestational age and parity (Figure 1b).

BP was measured in duplicate using a standard mercury sphygmomanometer and the average of two readings taken. All readings were taken by the same investigator (AK) with the subject in the sitting position. Korotkoff sounds 1 and 5 were used to define systolic and diastolic BP respectively. Mean BP was calculated as diastolic BP+^1^/_3_ pulse pressure. PE was defined according to the guidelines of the International Society for the Study of Hypertension in Pregnancy [31]. Diagnosis required two recordings of diastolic blood pressure ≥90 mm Hg, at least four hours apart; *or* one recording of diastolic BP≥120 mm Hg, in a previously normotensive woman; *and* urine protein excretion ≥300 mg in 24 hours, *or* two readings of ++ or more on dipstick analysis of a midstream or catheter specimen of urine, if no 24 hour collection was available.

Severe pre-eclampsia was defined as severe hypertension (diastolic blood pressure ≥110 mmHg) and mild proteinuria, or mild hypertension and severe proteinuria (a 24-hour urine sample that contained ≥3.5 g protein or a urine specimen ≥3+ protein by dipstick measurement). Patients with an abnormal liver function test (aspartate aminotransferase >70 IU/L) and thrombocytopenia (platelet count <100,000/cm^3^) were also classified as having severe pre-eclampsia. Gestational hypertension (GH) was defined as a diastolic blood pressure ≥90 mm Hg on at least two consecutive occasions in the second half of pregnancy, without proteinuria, in a previously normotensive woman [32]. Fetal growth restriction (FGR) was defined as birth weight less than the 5^th^ centile for gestational age. The controls consisted of 80 normotensive women matched for maternal age (±3 years) and parity (none or one to two deliveries).

We collected blood samples at similar gestational periods (±4 days) from the hypertensive and matched control participants. All women in the control group had uncomplicated pregnancies. They had no history of cardiovascular disease, hypertension, proteinuria or fetal growth restriction, and were not taking medication that could affect blood pressure. The diastolic blood pressure of all women with PE or gestational hypertension was higher than 95 mm Hg. They received oral antihypertensive therapy in the form of alpha methyldopa 750–1500 mg/day for clinical indications according to local clinical protocols. In accordance with local protocols, co-existing fetal growth restriction did not influence the decision to institute antihypertensive therapy.

Venous blood was collected before and after (24–48 hours) antihypertensive therapy was commenced. A single venous blood sample was collected from controls. The samples were centrifuged at 3,000 rpm for ten minutes. The serum was separated and frozen at −80°C for subsequent analysis.

Placental samples were collected from another group of women (n = 48) undergoing cesarean section (CS) before the onset of labour, including 14 presenting with PE, 10 with gestational hypertension (GH) and 24 normotensive controls matched for gestational age, maternal age (±3 years) and parity (none or one to two deliveries), and who were delivered by CS for obstetric reasons other than hypertension (e.g. preterm labour with an abnormal presentation or breech presentation at term). The hypertensive group included 12 women who received antenatal antihypertensive therapy (alpha methyldopa 750–1500 mg/day). They were matched for gestational age with 12 women who were not taking antihypertensive treatment. Four to five placental biopsies were obtained at random from the maternal surface of the placenta, free of placental membranes.

### Serum Assays

Using commercially available kits for the measurement of human sFlt-1, PlGF, free VEGF and sEng (R & D Systems, Minneapolis, Minnesota, USA), two-site enzyme linked immunosorbent assays (ELISAs) were conducted in duplicate according to the manufacturer's protocol. The minimum detectable levels for serum sFlt-1, PlGF, VEGF and sEng were 5 pg/ml, 7 pg/ml, 9 pg/ml and 7 pg/ml respectively. The VEGF level in the serum samples was below the detectable levels. Intra- and inter-assay co-efficients of variation respectively in our laboratory were as follows: serum sFlt-1 7%, 9%; PlGF 5%, 6%; and sEng 6%, 8%.

### Placental protein extraction and assays

The samples (placental villi, 164–559 mg wet weight) were rinsed in sterile phosphate buffered saline and snap frozen. A known weight of placental biopsy was homogenized manually in four volumes (w/v) of Tris buffered saline containing EDTA-free serine-/cysteine-protease inhibitor cocktail, diluted according to the manufacturer's instructions (Roche Biochemicals, Indiana, USA) using a homogenizer. The protein extract was collected after centrifugation (3,000 rpm for 10 minutes) and stored at −80°C until quantitative analyses were performed in batches.

The total protein concentration was determined with the Bradford assay (Pierce, Lausanne, Switzerland) using human serum albumin as a standard. Commercial ELISAs from R&D systems (Minneapolis, Minnesota, USA) were used to measure sFlt-1, PlGF, sEng and ‘free’ VEGF in placental extracts according to the manufacturer's protocol. All samples were assayed in duplicate and the intra- and inter-assay variations were <12% for all assays. The minimum detectable limits for the assays were: sFlt-1: 31.3pg/ml; PlGF: 3.9 pg/ml; sEng: 62.5 pg/ml; and free VEGF 7.8 pg/ml. These assays were validated for placental samples. Placental samples from cases with PE were diluted parallel to the standard curve in the sFlt-1, PlGF, sEng and VEGF ELISAs.

### Uterine artery Doppler measurements

Uterine artery Doppler pulsatility index (PI) was measured in the hypertensive and control groups at the time of recruitment. In women who received antihypertensive therapy, Doppler measurements were taken before and after (24–48 hours) therapy was initiated. The uterine artery was identified by a combination of real-time and colour Doppler techniques (iU22 Ultrasound System, Philips Medical Systems, Bothell, WA, USA). Blood velocity waveforms were recorded by the pulsed Doppler method (3.5 MHz curved probe; 120 Hz high-pass filter). Five consecutive flow velocity waveforms of good quality were recorded, and the mean pulsatility index (PI) derived.

### Statistical analysis

D'Agostino and Pearson Omnibus test was used to assess normality of continuous data. Analysis of variance (ANOVA) with Bonferroni post hoc tests were carried out to study the differences among the three groups. Data were analyzed in two gestational age intervals: <34 weeks representing early-onset disease and ≥34 weeks representing late-onset disease. Paired *t* tests were used to compare marker levels before and after antihypertensive therapy. The data were normally distributed after logarithmic transformation. Pearson correlation analysis was carried out to investigate the relationship between the parameters measured. Data were analyzed using SPSS® (SPSS version 15, 2007, SPSS Inc., Chicago, Illinois, USA). GraphPad Prism® 5.0 for Windows (InStata, GraphPad Software Inc., San Diego, California, USA) was used to test the normality of data. Results were considered statistically significant at *P*<0.05.

## Results

Figures 1 (a) and (b) are flow diagrams of the women recruited to the serum and placental arms of the study respectively. The baseline characteristics, mean blood pressure and uterine artery mean pulsatility index of the serum study groups are shown in Table 1. The time interval between serum sampling in the hypertensive women is also shown in Table 1. Among the 51 women with pre-eclampsia in this arm of the study, 16 (31%) had associated fetal growth restriction (FGR) and 8 (16%) had severe PE. All the severe PE cases were in the early-onset group.

**Table 1:** Baseline characteristics of the study groups in whom serum levels of angiogenic factors were measured, according to gestational age at recruitment.

	<34+0 weeks				≥34+0 weeks			
	Controls	PE	GH	*P* value	Controls	PE	GH	*P* value
n	41	28	13		39	23	16	
Age (years) †	31±4	30±5	32±4	0.6	31±4	30±4	32±5	0.2
BMI (kg/m^2^) †	27±4	30±4	27±4	0.07	28±3	27±5	29±5	0.2
Nulliparity [n (%)]	25 (61)	20 (71)	7 (54)	0.6	21 (53)	16 (70)	9 (56)	0.3
Current smoker [n (%)]	1 (2)	0 (0)	0 (0)	1	2 (5)	1 (4)	0 (0)	1
Caucasian [n (%)]	20 (48)	13 (46)	7 (54)	0.7	23 (59)	13 (57)	9 (56)	0.4
GA at recruitment (days) †	30±1.3	30±0.4	30.4±0.8	0.5	36.6±2.4	36±2.3	36.4±2	0.4
GA at delivery (days) †	39.7±2.3	33±1.7	36.7±2.9	<0.001	39.8±1.6	37.3±2	38.6±2.3	<0.001
Birth weight (grammes) †	3398±529	1685±204	2725±198	<0.001	3405±526	2896±689	3174±591	<0.001
Mean BP (mmHg)	85±12	126.6±12	125.1±12	<0.0001	85±11	121.1±11.1	114.5±6.1	<0.0001
Mean Pulsatility Index (PI)	0.76±0.1	1.51±0.5	0.84±0.1	<0.0001	0.59±0.1	0.85±0.2	0.61±0.1	<0.0001
Interval between samples (hours)		32±4	34±3			38±6	39±7	

BMI = body mass index.

GA = gestational age.

Mean BP = mean blood pressure.

PE = pre-eclampsia.

GH = gestational hypertension.

† Data presented as mean±SD and analyzed by one-way ANOVA with Bonferroni post hoc analysis.

### Serum levels prior to treatment

**Figure 2:**
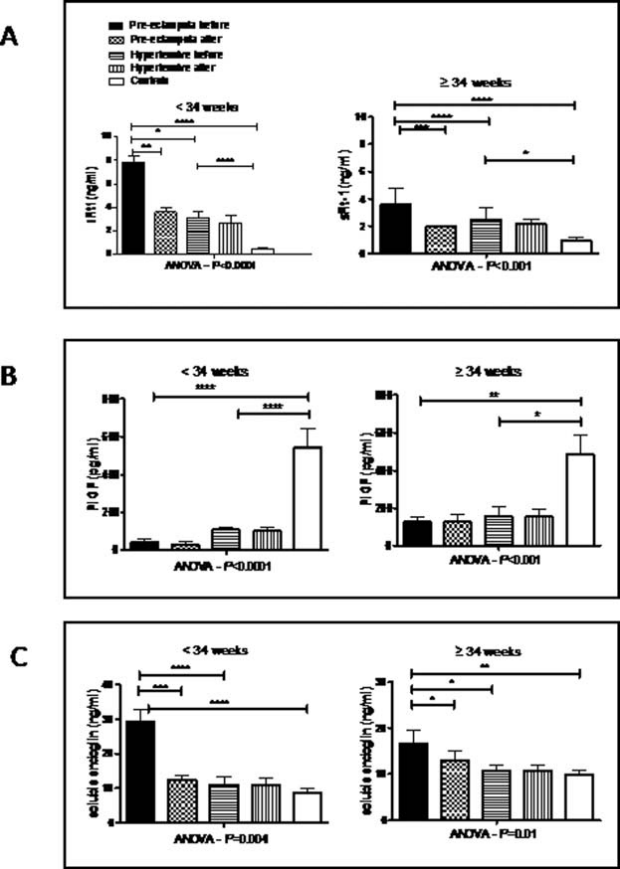
Serum concentrations of angiogenic factors in normotensive women, women with pre-eclampsia and women with gestational hypertension who received methyldopa. Mean serum sFlt-1 (a), PlGF (b) and sEng (c) concentrations in normotensives (controls), women with pre-eclampsia and women with gestational hypertension according to gestational age (GA) interval [early onset <34 weeks (41 controls, 28 PE, 13 GH) and late onset ≥34 weeks (39 controls, 23 PE, 16 GH)]. Error bars represent standard errors. Comparison of controls and cases (with PE or GH) was performed after logarithmic transformation. Levels before and after alpha methyldopa therapy are shown for women with pre-eclampsia and women with gestational hypertension. The levels in the three groups were compared using ANOVA with Bonferroni Dunn's posthoc tests. The levels before and after antihypertensive therapy are compared using the paired *t* test. ****P<0.0001, ***P<0.001, **P<0.01, *P<0.05. *P* values are shown only for differences which are statistically significant.

Figure 2 shows serum levels of angiogenic markers before and after antihypertensive therapy. Prior to treatment in women with PE, serum levels of sFlt-1 (Figure 2a) were significantly higher than normotensive controls (*P* <0.0001 both before 34 weeks and ≥34 weeks), and higher than women with GH (before 34 weeks, *P*<0.05; ≥34 weeks, *P*<0.0001). Serum sFlt-1 levels were also higher in GH compared with controls (before 34 weeks, *P*<0.0001; ≥34 weeks, *P*<0.05). Figure 2b shows that, prior to treatment in women with PE, serum PlGF levels were significantly lower than in controls (before 34 weeks, *P*<0.0001; ≥34 weeks, *P*<0.01) but not significantly different from women with GH. Levels in GH were also significantly lower than in controls (before 34 weeks, *P*<0.0001; ≥34 weeks, *P*<0.05). Figure 2c shows that, prior to treatment in women with PE, serum sEng was significantly higher than in normotensive controls (before 34 weeks, *P*<0.0001; ≥34 weeks, *P*<0.01), and higher than women with GH (before 34 weeks, *P*<0.0001; ≥34 weeks, *P*<0.05). Serum levels of sEng were not significantly different between GH and normotensive controls.

**Figure 3:**
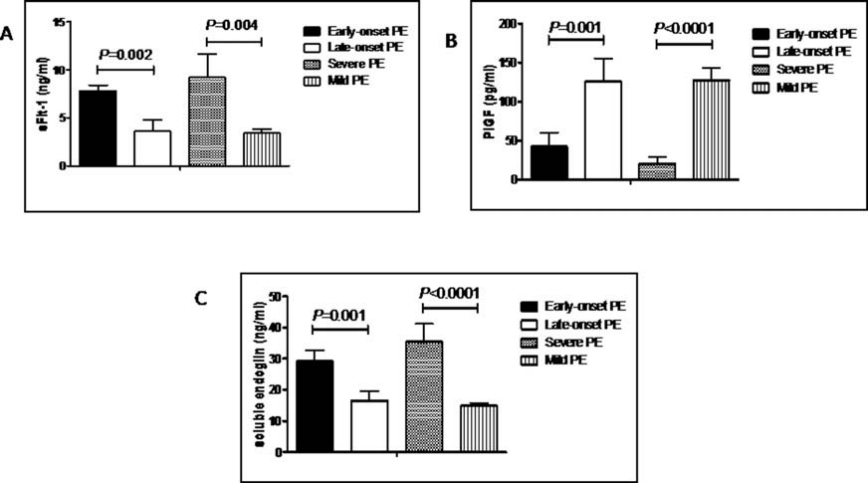
Maternal serum concentrations of angiogenic factors in early and late onset PE, and in mild and severe PE. Mean maternal serum concentrations of sFlt-1 (a), PlGF (b) and soluble endoglin (c) in women with early onset and late onset PE, and in women with mild and severe PE. Error bars represent standard errors. Early onset were compared with late onset, and mild with severe PE after logarithmic transformation using unpaired *t* test.

Figure 3 shows serum levels of these markers in early compared with late onset, and mild compared with severe pre-eclampsia. Serum levels of sFlt-1 were higher in early-onset compared with late-onset (*P* = 0.002), and in severe compared with mild pre-eclampsia (*P* = 0.004). Similarly, serum levels of sEng were higher in early-onset compared with late-onset (*P* = 0.001), and in severe compared with mild pre-eclampsia (*P*<0.0001). Serum PlGF was lower in early-onset compared with late-onset (*P* = 0.001), and in severe compared with mild pre-eclampsia (*P*<0.0001).

### Effect of treatment on serum levels

Figure 2 shows the effect of treatment with methyldopa on serum levels of sFlt-1, PlGF and sEng. In women presenting with PE, antihypertensive treatment was associated with a significant fall in the serum concentrations of both sFlt-1 (before 34 weeks, *P*<0.01; ≥34 weeks, *P*<0.001) and sEng (before 34 weeks, *P*<0.001; ≥34 weeks, *P*<0.05) (Figures 2 a and c respectively). Antihypertensive therapy had no significant effect on serum levels of PlGF in women with PE (Figure 2b), or on the level of any of these proteins in women with GH.

### Placental levels in hypertensive disorders

**Figure 4:**
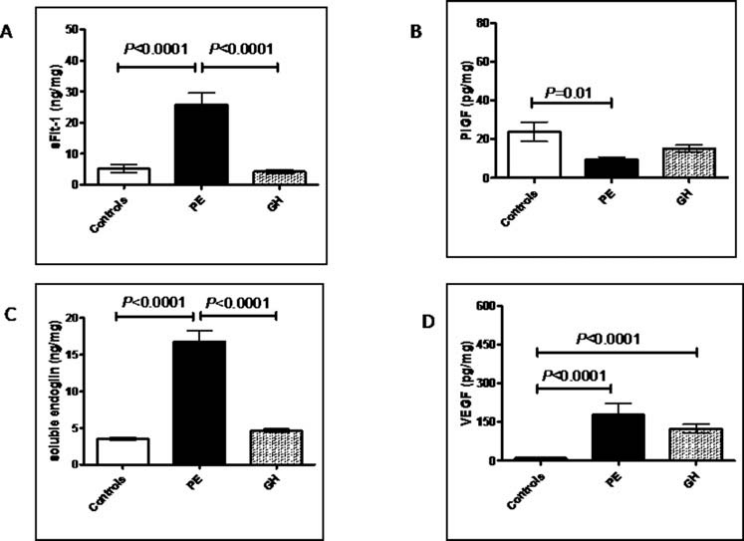
Placental concentrations of angiogenic factors in normotensive women, women with pre-eclampsia and gestational hypertension. Concentrations of sFlt-1(a), PlGF (b), soluble endoglin (c) and VEGF (d) (expressed per mg protein) in placental tissue from normotensive (controls, n = 24, pre-eclampsia (PE, n = 14) and gestational hypertension (HT, n = 10 pregnancies. Error bars represent standard errors. Comparison of controls and cases (with PE or GH) was performed after logarithmic transformation. The mean gestational ages (days) for the three groups were (mean±SD): controls 242±16; pre-eclampsia 238±13; gestational hypertension 243±20.

The results of the placental analysis are presented in Figure 4 and Table 2. Figure 4 shows the placental extract concentrations of sFlt-1, PlGF, sEng, and VEGF in 24 controls, 14 PE cases and 10 GH cases. Placental levels of sFlt-1 were significantly higher (5-fold) in PE compared to GH (*P*<0.001) or controls (*P*<0.001). There was no significant difference in sFlt-1 levels between women with GH and controls (Figure 4a). Placental levels of PlGF were significantly lower (*P* = 0.01) in PE compared to controls (Figure 4b). Placental levels of PlGF were lower in GH compared with controls but this difference did not achieve statistical significance (*P* = 0.5). Placental concentrations of sEng were significantly higher (*P*<0.0001) in women with PE compared to GH or normotensive controls (Figure 4c). There was no significant difference in placental sEng between controls and women with GH (*P* = *0.07*). Placental VEGF was significantly higher in women with either PE or GH (*P*<0.0001) compared with normotensive controls (Figure 4d). There was no significant difference in placental VEGF between women with PE and women with GH (*P* = 0.6).

**Table 2:** Antihypertensive therapy and placental concentrations of angiogenic factors.

	PE			GH			Controls
	Anti-hypertensive (n = 7)	No anti-hypertensive (n = 7)	*P* value	Anti-hypertensive (n = 5)	No anti-hypertensive (n = 5)	*P* value	
sFlt-1 (ng/ml)	17.7 (3)*	33.9 (5)*	0.01	3.1 (0.4)	5.3 (0.8)	0.06	5.2 (1.2)
PlGF (pg/ml)	8.4 (1.8)*	7.7 (2)*	0.56	20.7 (3)	15.9 (4)	0.18	23.9 (5)
sEng (ng/ml)	13.1 (1.6)*	19.6 (1.7)*	0.02	3.8 (0.4)	4.3 (0.3)	0.35	3.5 (0.2)
VEGF (pg/ml)	107.5 (30)*	247 (82)*	0.09	108 (39)*	166 (32)*	0.1	10.4 (1.2)

Placental concentrations of sFlt-1, PlGF, sEng and VEGF (expressed per mg protein) in normotensive controls, pre-eclampsia (PE) and gestational hypertension (GH), grouped according to whether they received antihypertensive therapy or not. The mean gestational ages for the three groups were (mean±SD): controls 242±16; pre-eclampsia 238±13; gestational hypertension 243±20. The *P* values represent the statistical difference between the groups with hypertension who received antihypertensive therapy or not.

PE = pre-eclampsia.

GH = gestational hypertension.

Data presented as mean (standard error of the mean), and analyzed by independent *t* test.

* *P*<0.05 compared with normotensive control group.

### Antihypertensive treatment and placental levels

Table 2 shows the placental concentrations of the same four markers in women with PE and gestational hypertension, grouped according to whether they received antihypertensive therapy (all with methyldopa) or not. In women with PE, treatment with methyldopa was associated with a significantly (almost 50%) lower placental concentration of sFlt-1 (*P* = 0.01). In women with GH, treatment was also associated with a lower placental sFlt-1 concentration but this did not achieve statistical significance (*P* = 0.06). Antihypertensive treatment was also associated with significantly (*P* = 0.02) lower placental sEng in women with PE, but not in gestational hypertension. Treatment with methyldopa did not affect placental levels of PlGF or VEGF.

There was no significant difference in mean uterine artery Doppler pulsatility index before and after methyldopa treatment, in either the PE (<34 weeks, *P* = 0.07; ≥34 weeks, *P* = 0.6) or GH group (<34 weeks, *P* = 0.18; ≥34 weeks, *P* = 0.6).

## Discussion

Pre-eclampsia remains one of the most complex disorders of human pregnancy. The lack of suitable animal models with placental features of the disease means that we have to rely, for the most part, on human studies. The maternal response to the presence of a pregnancy and placental activity remain the focus of research into the disease. The data from this study confirm that, in both early and late onset PE, maternal serum levels of sFlt-1 and sEng are higher, and PlGF lower, in women presenting with PE [1]–[3]. In addition, we found that placental sFlt-1 and sEng were significantly increased, and PlGF decreased, in women with PE compared to controls.

Our data suggest that, in pre-eclampsia, placental concentrations of sFlt-1, sEng and PlGF mirror the maternal serum changes. These findings are consistent with the view that the placenta is the main source of sFlt-1, sEng and PlGF during pregnancy [9].

Circulating sFlt-1 can bind to PlGF and VEGF, effectively inhibiting their actions [11], [33]. Soluble Flt-1 is therefore considered to be a circulating anti-angiogenic factor. In our study, as previously described, levels of sFlt-1 were elevated and PlGF reduced in the serum of women with PE prior to treatment [1], [4]. The lower levels of free PlGF found in the serum of women with PE may be the result of impaired placental production or secretion, or due to increased binding by sFlt-1 in maternal serum.

Our findings indicate that antihypertensive treatment with alpha methyldopa is associated with a significant fall in *serum* concentrations of both sFlt-1 and sEng in women presenting with either early onset or late onset PE. Methyldopa therapy had no significant effect on the *serum* levels of these markers in women presenting with gestational hypertension. Consistent with the trend in maternal serum, antihypertensive treatment with methyldopa was also associated with significantly lower *placental* concentrations of both sFlt-1 and sEng in PE, but not in gestational hypertension. These findings suggest that, in pre-eclampsia, alpha methyldopa may have a direct effect on placental synthesis and/or secretory functions and that this effect may not be simply the result of a reduction in maternal blood pressure and/or a change in utero-placental blood flow. However, sFlt-1 and sEng are also produced by vascular endothelial cells and we cannot exclude an endothelial cell effect of the medication in women with PE. The specific effect in PE with no effect in GH indicates that methyldopa has a different effect on placental and/or endothelial production and/or secretion of angiogenic factors depending on the pathophysiology of the hypertensive disorder. These findings support the concept of a fundamental difference in pathophysiology between gestational hypertension and the pathological endothelial toxic effect of pre-eclampsia.

Alpha methyldopa acts on α_2_-adrenergic receptors, primarily in the central nervous system (CNS) although an effect on peripheral α_2_-adrenoreceptors may also play a part [34]–[35]. Its main active metabolite is alpha-methyl norepinephrine, which resembles norepinephrine in its effects. Stimulation of pre-synaptic α_2_-adrenoreceptors in the CNS leads to a reduction of central sympathetic outflow. This causes a reduction in blood pressure [36]. α_2_-adrenoreceptors have also been identified in a variety of other human tissues outside the CNS, including myometrium and placenta [37]–[38]. An almost universal effect of α_2_-adrenoreceptor stimulation is the inhibition of adenylyl cyclase which leads to decreased production of cAMP [39]–[40]. cAMP has been shown to be a strong inducer of Flt-1 expression in mice [41]–[42].

In 2007, Muthig et al demonstrated that down-regulation of α_2β_-adrenoceptors in mice placenta resulted in increased levels of Flt-1 and sFlt-1, [43] suggesting that stimulation of α_2β_-adrenoceptors can suppress production of sFlt-1. Deletion of the gene encoding α_2β_-adrenoceptors resulted in upregulation of Flt-1 in spongiotrophoblast cells. These data support a direct link between adrenergic receptor signalling and angiogenic regulation by the VEGF system. This may be the mechanism by which alpha methyldopa leads to the reduction in sFlt-1 which our data support. Although this study [43] was done in mice, several functionally relevant polymorphisms that may potentially affect sFlt-1 expression and blood vessel formation have been identified in human adrenoceptor genes. This adds weight to the argument that methyldopa has an effect on maternal production of vasoactive substances: the fact that we see a different response in women with pre-eclampsia may reflect the finding that women with this disease are producing abnormal amounts of these substances in the first place.

Due to the rapid evolution of hypertensive diseases in our study groups, we could investigate only the biological effects of the antihypertensive treatment over a short time interval (maximum 48 hours). Compared to long-term studies in non-pregnant women [30], studies during pregnancy are limited by the fact that it is not possible to analyze the placenta before and after initiating treatment. Thus we decided to compare women with hypertensive disorders receiving methyldopa with women with hypertensive disorders not receiving treatment. Clinically, the need for antihypertensive treatment is a marker of disease severity; thus, prior to treatment, higher levels of sFlt-1 and sEng would be expected in the treatment group compared with the non-treatment group. Nevertheless, we found that antihypertensive treatment was associated with significantly lower levels of these two markers in the placenta of women treated with methyldopa compared to the placenta of untreated women.

A potential limitation of our study is the short time interval (24 to 48 hours) from initiation of antihypertensive treatment to venous blood sampling. It would be interesting to investigate the effect on angiogenic markers levels at longer intervals, e.g. a week after starting treatment. However, most women with hypertensive disorders in pregnancy, and particularly PE, will need delivery soon after starting antihypertensives, such that long-term follow-up is often precluded.

Our findings suggest that any future research into the use of serum markers to screen or monitor hypertensive disorders of pregnancy should take account of possible effects of antihypertensive therapy on marker levels. Further research is needed to evaluate whether different antihypertensive drugs have different effects on anti-angiogenic factors. Such research will improve our understanding of the pathophysiology of pre-eclampsia but may also lead to better therapeutic clinical protocols. Raised maternal serum levels of sFlt-1 can be detected several weeks prior to the onset of clinical pre-eclampsia. It is worth investigating whether administration of α-methyldopa at this point might have an effect on levels of anti-angiogenic factors and modify the disease process. Our findings also have potential implications outside the specialty of obstetrics. Women who develop pre-eclampsia are at significantly increased risk, later in life, of cardiovascular disease such as ischemic heart disease and stroke. Within this context, it is not known whether the use of specific antihypertensive drugs can also have a long-term beneficial effect. Furthermore, it remains to be determined whether the use of these antihypertensive drugs outside pregnancy could have a similar beneficial effect on anti-angiogenic factors and subsequently translate into clinical benefit. We hope that our data will stimulate further research in these areas.

It is not yet clear whether sFlt-1 and sEng are directly involved in the pathophysiology of PE or are simply markers of the disease process. Our data showing that antihypertensive treatment with alpha methyldopa is associated with a significant fall in their concentrations in both maternal serum and placenta is consistent with a positive effect on the control of disease progress. This finding supports the concept that pre-eclampsia combines an excessive maternal response to the presence of a pregnancy and placenta and progressive utero-placental insufficiency during the second half of pregnancy at the time of maximal fetal growth.
